# The Onion

**Published:** 1888-12

**Authors:** 


### THE ONION.

No family ought to be without onions the whole year round. Plant old onions in the fall, and they will come up at least three weeks earlier in the spring than by spring planting. Give children of all ages a few of them raw, as soon as they are fit to be eaten ; do not miss treating them with a mess of raw onions three or four times a week. When they get too large and strong to be eaten raw, then boil or roast them. During unhealthy seasons, when diphtheria and like contagious diseases prevail, onions ought to be eaten in the spring of the year, at least, once a week. Onions are invigorating and prophylactic beyond description. Children do not die of diphtheria or scarlatina, anginosa, etc., where onions were freely eaten.

